# Trends in health service needs, utilization, and non-communicable chronic diseases burden of older adults in China: evidence from the 1993 to 2018 National Health Service Survey

**DOI:** 10.1186/s12939-023-01983-7

**Published:** 2023-08-30

**Authors:** Nina Wu, Xueqin Xie, Min Cai, Youli Han, Shiyong Wu

**Affiliations:** 1 Department of Health Management and Policy, School of Public Health, Capital Medical University, No. 10 Xitoutiao, Youanmenwai Street, Fengtai District, Beijing, 100069 China; 2 Centre for Health Statistics Information, National Health Commission of the People’s Republic of China, No. 1, Xizhimenwai South Road, Xicheng District, Beijing, 100044 China

**Keywords:** Older adult, Health service needs, Health service utilization, Non-communicable chronic diseases, China

## Abstract

**Background:**

Aging is associated with an increased prevalence of non-communicable chronic diseases (NCDs), functional impairments, and diverse demands for health services. This study analyzed the trends in older adults’ needs and utilization of health services from 1993 to 2018 in China, as well as chronic disease-related economic burdens.

**Methods:**

The research data were collected from the six cross-sectional National Health Service Survey (NHSS), implemented every 5 years from 1993 to 2018. A multi-stage stratified random cluster sampling method has been adopted in the NHSS. The data on the older population's socio-economic characteristics, health service needs, and utilization were collected from the 6 waves National Health Service Survey (NHSS) 1993–2018. In the 2013 and 2018 NHSSs, EQ-5D-3L and visual analogue scale were used to evaluate the health condition. And the prevalence of NCDs and related Out-of-pocket (OOP) expenditures were collected. Functional dependency and impairment were collected in 2018. The Katz Activities of Daily Living scale was used to evaluate six functions, including self-feeding, dressing, bathing, transferring, toilet hygiene, and controlling bowel movements.

**Results:**

The two-week morbidity rate and prevalence of NCDs showed a rapid upward trend in older adults. With the development of health system reform and universal health insurance coverage, older adults' two-week medical consultation rate increased from 25.6% in 1993 to 40.1% in 2018, and the hospitalization rate rose from 6.1% to 24.9%. The difference in health service needs and utilization between urban and rural areas decreased, and the hospitalization rate in rural areas (26.3%) exceeded that in urban areas (23.6%) for the first time in 2018. Functional independence become more severe as aged. The proportion of severe functional impairment was 6.9% and 2% in the group aged 80 or over and group 70–79 years, respectively. Regarding disability status, 32.5% had hearing problems and 31.4% had visual impairment. The highest prevalence rates of NCDs in older adults were found in hypertension (36.9%), followed by diabetes (10.6%), cerebrovascular disease (5.4%), ischemic heart disease (4.5%), and intervertebral disc disease (4.2%). The average annual OOP expenditures attributed to NCDs increased from ¥2481.8 RMB in 2013 to ¥8255.9 RMB in 2018 for older adults. About 90.7% of older adults prefer to live in the residential community, leading to the demands for preventive healthcare (30.4%), medical treatment (14.1%), and elderly education (8.6%).

**Conclusion:**

The elevated risks of age-related impairments and chronic morbidities, and increased demands for preventive healthcare are critical public health issues. Policymakers should strengthen primary healthcare and move towards integrated delivery to improve access and quality of care for older adults. The integration of healthcare and social security constitutes an adaptive trend in meeting the multi-level demands of an aging society.

## Introduction

The elderly population is rising rapidly worldwide, augmenting the challenges to healthcare financing and delivery. In May 2020, the UN General Assembly declared 2021–2030 the decade of healthy aging, highlighting the importance for policymakers across the world to focus policy on improving the lives of older people. China has the world's largest older population and the proportion of adults over 60 years have risen rapidly over the past 30 years. According to the China Demographic Yearbook, the size of the older adult aged above 60 grew from 110 million in 1993, accounting for 9.5% of the total population, to 250 million, accounting for 17.9% of the total population in 2018. China’s aging trends show two characteristics. First, the growth rate for the rural population of older adults was higher than that for the urban population. In 1993, the number of older adults accounted for 8.9% of the population in rural areas. By 2018 it had increased to 20.5%. Second, the proportion of adults over 80 also gradually increased. In 1993, the proportion of individuals over 80 in China was 0.8%, and by 2018 it increased to 2.1%. The senior elderly may face more serious health problems.

The socioeconomic progress, urbanization, and China's demographic transition leads to new healthcare challenges in the past 3 decades. Major healthcare challenges include the management of NCDs, physical frailty, neurodegenerative diseases, cardiovascular diseases, and the growing necessity to provide palliative care for the elderly [1]. These chronic conditions are complex to treat and manage and are associated with increased rates of functional disability and increased family-based care and social care needs. But personnel planning in geriatric and rehabilitation medicine has not kept pace with the growth of the elderly population, and there is a shortage of healthcare resources for the aging population, especially in rural China. Substantial gaps are apparent in rural versus urban residents in health equity and socioeconomic status [2].

In 2000, the New Rural Cooperative Medical Scheme was set up to provide public health insurance for rural residents. In 2009, China launched a major healthcare reform and the government has since quadrupled its funding for health. The reform's first phase (2009–2011) emphasized expanding social health insurance and primary healthcare coverage for all and strengthening infrastructure. The second phase (2012–2020) prioritized reforming its healthcare delivery and provider payment system [3, 4]. The 13th Five-Year Plan for Healthy Ageing, the first national policy focused on healthy aging was released by the Chinese Central Government in 2017, signaling healthy aging as a key priority in the national political agenda. This plan elaborated on the vision of a consolidated policy system centered on the national healthy aging plan and formulate a national implementation work plan to promote an integrated healthcare model for older people.

The World Health Organization (WHO) recommended a societal approach to population aging that requires a transformation of health systems away from disease-based curative models and toward the provision of integrated care centered on the health service demands of older individuals. According to the WHO’s research, the greatest costs to society are not the expenditures made to foster this functional ability, but rather the benefits that might be missed if we fail to deliver the appropriate adaptations and investments [5]. Although a strong political commitment to action on healthy aging has been made over the past several years, many policy gaps remain in financing and integrated healthcare systems [6–8]. In recent years, studies on the issue of trends in aging and rising in related health service utilization of older adults have increased rapidly [9, 10]. Previous studies have described the aging trends and the high economic burden of older adults [11–13]. In particular, reform effects in some pilot areas, such as universal health insurance and primary health services, were evaluated [14–16]. However, there is a lack of research on the utilization of health services by the elderly with aging trends at the national level, as well as the disparity of health service needs and utilization between urban and rural areas.

Therefore, it is necessary to study the trends in the health service needs and utilization of the elderly in the aging process to provide a basis for the corresponding adjustment of the service model. This study provides a systematic evaluation of the trends of health service needs and utilization in the past 3 decades national wide, and analyzes functional independence and NCDs burden with respect to the elderly, which provides a comprehensive perspective on the health policies in China and is invaluable to international research on aging and health service research.

## Methods

### Sampling methods and data sources

The research data were collected from the six waves National Health Service Survey (NHSS) implemented every 5 years from 1993 to 2018. A multi-stage stratified random cluster sampling method has been adopted in the NHSS. From 1993 to 2008 NHSSs, 92–94 counties (cities or districts) were randomly selected from the country’s 31 provinces, autonomous regions, and municipalities. From the 2013 NHSS, the number of counties increased to 156. In each county, there were 5 sample townships/neighborhoods, within which, two administrative villages /neighborhood committees were collected. Furthermore, 60 households from each sample village/ neighborhood committee were randomly selected. The number of 6 waves NHSSs sampled populations were 214,844, 215,506, 193,689, 177,501, 273,688, and 256,304, separately.

According to the definition of the elderly population by the China Bureau of Statistics and the health welfare policy, the subjects of this study included the elderly aged 60 and above. Finally, the number of elderly adults from the NHSSs data was 26,970 in 1993, 26,261 in 1998, 29,559 in 2003, 29,634 in 2008, 61,057 in 2013, and 69,342 in 2018, accounting for 12.6%, 12.2%, 15.3%, 16.7%, 22.3%, and 27.1% of the sampled populations. The primary instrument was questionnaires that encompassed health status, health service utilization, and out-of-pocket health expenditures.

### Principal variables

#### Health service needs and utilization

The health service needs of older adults were measured by two-week morbidity and the prevalence rate of NCDs. The denominator is the number of elderly people surveyed. The numerator is the number of patients within two weeks and those with NCDs. The NCDs that had been diagnosed by doctors were self-reported by the respondents. Health service utilization of older adults included two-week medical consultation rates and annual hospitalization rates. We reported the trends of health service needs and utilization from 1993 to 2018.

#### Health condition

EQ-5D-3L was used in 2013 and 2018 NHSS for self-assessed health problems, including the following five dimensions: mobility, self-care, usual activities, pain/discomfort, and anxiety/depression. Each dimension has 3 levels: no problems, some problems, and extreme problems. The EQ visual analogue scale (VAS) records the respondents’ self-rated health on a horizon visual analogue scale where the endpoints are labeled ‘Best imaginable health state (score 100)’ and ‘Worst imaginable health state (score 0)’. We reported the results in 2013 and 2018 of different aged groups to show that health problem change with age.

#### Functional independence and impairment

The 2018 NHSS added an elderly questionnaire for subjects aged 60 and above, including the Katz Activities of Daily Living (ADL) scale, and functional impairment of hearing, vision, and intelligence. The Katz ADL scale was used to evaluate six functions, including self-feeding, dressing, bathing, transferring, toilet hygiene, and controlling bowel movements. The subjects scored yes/no for independence in each of the six functions. A total score of 6 indicated full function, 4–5 indicated light impairment, 2–3 indicated median impairment, and 0–1 indicated severe functional impairment. We reported the functional independence and impairment in 2018 of different aged groups to show the health problems of the oldest-old adults.

#### Health service utilization and economic burden of NCDs

NCDs that doctors had diagnosed were self-reported by the respondents and was multiple-choices question. The trained investigator recorded the self-reported diagnosis of the respondents and recorded the code according to the NHSS disease coding manual. From the 2013 NHSS, the out-of-pocket (OOP) expenditures was surveyed to evaluate the disease economic burden of individuals, which included direct medical costs paid by individuals and indirect costs, such as transportation and meals costs. We reported the prevalence rates, hospitalization rates, and OOP expenditures of common NCDs. The value in 2018 was converted to the 2013 value using the local GDP deflator.

## Results

### Rising needs for health services in older adults

From 1993 to 2018, the two-week morbidity rate of older adults continued to increase, especially in 2008 and 2018, increasing by approximately 30%. From 1993 to 2018, the prevalence rate of NCDs in older adults increased from 50.6% to 59.2% (Table 1). The two-week morbidity rate and the prevalence rate of NCDs in older adults living in urban areas were higher than those of the rural population, but the difference between urban and rural areas decreased.

**Table 1:** Two-week morbidity rates and prevalence of NCDs of older adults from 1993 to 2018 (%)

**Variables**	**1993**	**1998**	**2003**	**2008**	**2013**	**2018**
Two-week morbidity rate						
Urban	31.0	36.4	36.8	53.4	49.1	57.3
Rural	21.6	24.5	29.4	37.8	36.8	53.4
Total	25.0	29.0	32.1	43.2	43.3	55.5
Prevalence rate of NCDs						
Urban	73.0	75.3	71.6	79.0	54.6	60.6
Rural	37.8	35.0	37.6	49.3	45.4	57.5
Total	50.6	50.2	50.1	59.6	50.2	59.2

### Rising outpatient and inpatient utilization in older adults

The utilization of health services by older adults has increased rapidly, especially in the past five years. The two-week medical consultation rate increased from 25.6% in 1993 to 40.1% in 2018. The hospitalization rate rose from 6.1% in 1993 to 24.9% in 2018, and the increase in the hospitalization rate was more pronounced in rural areas higher than that in urban areas. In 2018, the hospitalization rate in rural areas (26.3%) exceeded those in urban areas (23.6%) for the first time (Table 2).

**Table 2:** The outpatient and inpatient utilization for the older-adult population from 1993 to 2018 (%)

**Variables**	**1993**	**1998**	**2003**	**2008**	**2013**	**2018**
**Two-week medical consultation rate**						
Urban	31.0	33.0	26.3	28.1	25.4	40.5
Rural	22.5	25.1	27.5	28.4	23.9	39.6
Total	25.6	28.0	27.1	28.3	24.7	40.1
**Hospitalization rate**						
Urban	8.7	11.9	11.0	17.2	18.9	23.6
Rural	4.6	5.5	5.7	12.0	16.8	26.3
Total	6.1	7.9	7.6	13.8	17.9	24.9

### The health condition in different age groups of older adults

The self-rated health score of older individuals decreased in 2018 (70.7) relative to 2013 (73.3). The health score of elderly people in each age group has reduced, with the lowest score being the group aged over 80, from 67.2 in 2013 to 66.5 in 2018. The health problems related to mobility, self-care, daily activities, pain/discomfort, and anxiety/depression of older adults became serious from 2013 to 2018. The proportion of older adults who had pain/discomfort increased from 25.5% in 2013 to 35.1% in 2018. The health problems grew in proportion with age, in older adults aged 80 or above, 39.2% and 42.5% had problems with mobility and pain/discomfort respectively (Table 3).

**Table 3:** Self-assessed health problems of older adults in different age groups, 2013 and 2018 (%)

**Health condition**	**2013**				**2018**			
	**60–69 (*N* = 35,963)**	**70–79 (*N* = 18,317)**	**80 + (*N* = 6,777)**	**Total (*N* = 61,057)**	**60–69 (*N* = 41,466)**	**70–79 (*N* = 20,456)**	**80 + (*N* = 7,420)**	**Total (*N* = 69,342)**
Mobility	9.1	19.8	38.2	14.9	15.2	25.0	39.2	20.3
Self-care	4.4	10.3	24.3	7.9	5.7	10.4	20.7	10.5
Usual Activities	6.8	15.3	33.9	11.8	9.9	16.8	31.6	13.9
Pain/Discomfort	21.1	30.1	38.6	25.5	32.2	38.7	42.5	35.1
Anxiety/Depression	7.8	10.7	15.9	9.5	11.8	13.6	14.8	12.6
Self-rated health score	75.0	71.0	67.2	73.3	72.2	68.8	66.5	70.7

### Functional independence and impairments

Functional independence become more severe as aged. The proportion of severe functional impairment was 6.9% and 2% in the group aged 80 or over and group 70–79 years, respectively. Regarding disability status, 32.5%, 31.4%, and 2.9% of older adults had hearing, visual, and intellectual impairment, respectively. The health problems deficits with respect to hearing, vision, and intellectual disability also increased significantly with age. For adults aged 80 or above, 57.1% had hearing impairment, 48.6% had vision impairment, and 7.8% had intelligence impairment. This demonstrates the impact of age, with those aged 80 or over showing a greater need for assistance than those aged 65–79 years (Table 4).

**Table 4:** Functional impairment and health service demands of older adults in different age groups, 2018 (%)

**Disability status and health service demands**	**60–69**	**70–79**	**80 + **	**Total**
**Functional independence**				
Light impairment	2.2	4.5	10.3	3.7
Median impairment	0.6	1.2	3.5	1.1
Severe functional impairment	0.9	2.0	6.9	1.8
**Functional impairment**				
Hearing	25.3	38.0	57.1	32.5
Vision	26.0	36.2	48.6	31.4
Intelligence	2.0	3.1	7.8	2.9
**Health service demands**				
Preventive Healthcare	29.4	33.0	28.1	30.4
Medical Treatment	13.8	15.0	13.6	14.1
Elderly Education	8.2	9.8	7.7	8.6
Cultural and Sports Activities	4.8	4.7	2.8	4.6
Living Care	2.3	2.7	3.5	2.5
Rehabilitation Nursing	1.9	2.1	2.2	1.9
Mental healthcare	1.9	1.8	2.1	1.8
Assistance in Renting Equipment	0.1	0.1	0.2	0.1
Others	2.9	3.0	3.0	2.9

In 2018, 90.7% of older adults preferred to live in the community, increasing demand for community-based or home-based elderly care services. When asked which health services had been used, the most common ones were preventive healthcare (30.4%), medical treatment (14.1%), elderly education (8.6%), cultural and sports activities (4.6%), and living care (2.5%). As individuals aged, we noted increases in their demands for rehabilitation nursing care, living care, mental healthcare, and assistance in renting equipment and/or products (Table 4).

### Health service utilization and economic burden of NCDs

As the people aged, they also became more likely to experience multiple chronic conditions and to need more medical services. In urban areas, the highest level of health service needs and utilization was recorded among adults aged over 80, among whom the prevalence of NCDs and hospitalization rates were 67.5% and 33.1%, respectively. In the rural area, the highest level of health service needs and utilization were among adults aged 70–79, among whom the prevalence of NCDs and hospitalization rates were 62.6% and 30.7%, respectively (Table 5).

**Table 5:** The health service needs and utilization by age group in urban and rural areas, 2018 (%)

**Variables**	**Urban**			**Rural**		
	**60–69 (*N* = 21,978)**	**70–79 (*N* = 11,177)**	**80 + (*N* = 4,351)**	**60–69 (*N* = 19,516)**	**70–79 (*N* = 9,232)**	**80 + (*N* = 3,088)**
Two-week morbidity rate	52.9	63.5	63.8	51.1	57.8	54.8
Prevalence of NCDs	55.8	67.3	67.5	55.0	62.6	58.2
Two-week medical consultation rate	36.3	48.0	47.5	38.6	43.5	37.7
Hospitalization rate	19.7	27.7	33.1	23.8	30.7	28.3

The highest prevalence rates with respect to NCDs in older adults were reflected by hypertension (36.9%), followed by diabetes (10.6%), cerebrovascular disease (5.4%), ischemic heart disease (4.5%), and intervertebral disc disease (4.2%) in 2018. The rates of hypertension (39.2%) and diabetes (13.5%) in urban areas were higher than in rural areas (hypertension, 34.1%; diabetes, 7.2%), while rates for cerebrovascular disease (6.2%) and intervertebral disc disease (5%) in rural areas were higher than in urban areas (cerebrovascular disease, 4.7%; intervertebral disc disease, 3.5%). In the spectrum of inpatient diseases, the hospitalization rate for cerebrovascular disease was the highest (2.7%), followed by hypertension (1.8%). Although the prevalence rate for chronic obstructive pulmonary disease (COPD) was 2.9% which was lower than that for the other major NCDs, the hospitalization rate was 1.4%, ranking third among NCDs hospitalizations. Intervertebral disc disease was related to disability risk in older adults, entering the list of the top five NCDs for the first time in 2018 (Table 6).

**Table 6:** Top six NCDs and the rates of inpatient admission, 2018 (%)

**NCDs**	**Urban**		**Rural**		**Total**	
	**Prevalence rate**	**Hospitalization rate**	**Prevalence rate**	**Hospitalization rate**	**Prevalence rate**	**Hospitalization rate**
Hypertension	39.2	1.8	34.1	1.9	36.9	1.8
Diabetes	13.5	1.1	7.2	0.9	10.6	1.0
Cerebrovascular disease	4.7	2.1	6.2	3.4	5.4	2.7
Ischemic heart disease	5.0	1.3	3.8	1.5	4.5	1.4
Intervertebral disc disease	3.5	1.1	5.0	1.3	4.2	1.2
Chronic obstructive pulmonary disease	2.4	1.2	0.9	1.8	2.9	1.4

The average annual OOP expenditures of NCDs was ¥8256 RMB, accounting for 13.6% of GDP per capita in 2018, a substantial increase from ¥2,481.8 RMB in 2013. The average annual growth rate of urban expenditures (38.1%) was greater than that of rural areas (30.1%), and the expenditures gap was also widening (Table 7).

**Table 7:** Average annual OOP expenditures of NCDs, 2018

Variables	2013			2018		
	**Urban**	**Rural**	**Total**	**Urban**	**Rural**	**Total**
Total annual OOP (RMB)	8987.7	6165.4	15,153.1	37,446.7	20,062.8	57,509.5
Number of older adults	32,031	29,026	61,057	36,734	32,924	69,658
Average annual OOP (RMB)	2805.9	2124.1	2481.8	10,194.0	6093.7	8256.0

The values in 2018 were converted to 2013 values using local GDP deflator (1.0675)

One US dollar equals to 6.617 RMB in 2018, and 6.193 RMB in 2013

## Discussion

The study used variables related to health from multiple perspectives, with a large sample size that could provide a comprehensive view of the health status and healthcare demands of older adults in China. In the 30-year-long NHSSs, the survey tools are also improving, several survey instruments were used in the 2013 and 2018 NHSS to evaluate the health status and problems, like EQ-5D-3L and ADL, that could provide more accurate evidence for analyzing the functional dependency status and long-term care needs of the elderly. The study examines the trends in older adults' health service needs and utilization from 1993 to 2018 and reveals the magnitude of the increase in the disease burden of NCDs among older adults at the national level, which would be of interest to researchers and policymakers concerned with the medical care of the elderly.

### There were persistent challenges for health financing with rising healthcare needs and expenditures

We currently classify older adults as primarily those individuals 60–69 years of age, but in the next 10–20 years the greatest proportion will be over 80 years of age. Degenerative diseases, functional impairments, and disability conditions in older adults have generated different priorities and new challenges to health financing. As people age, more likely they are to experience multiple chronic conditions. As a result, the impact of multiple morbidities on functioning, quality of life, and mortality risk may be significantly greater than the sum of the individual effects that might ordinarily be expected from these conditions [17].

Our study revealed that older individuals experienced a higher economic burden with regard to NCDs. The OOP expenditures and growth rate of urban elderly patients with chronic diseases are higher than those of rural elderly. Previous research showed that universal health insurance had not narrowed the urban–rural inequality in health care access and fiscal spending [18]. This may also be related to the difference in welfare systems, and social-economic development between rural and urban [19]. While health insurance covered major diseases for this population, attention needs to be allotted to the economic burden placed on older patients with NCDs who take medications over extended periods. Therefore, the health financing and public health insurance system should adapt to the affordable and accessible health service demands of older adults [20, 21].

### Older adults have various demands for home-based and community-based health services

The study indicated substantially increased demand for health services, and greatly increased hospitalization rates in rural areas, which exceeded that of urban areas for the first time in 2018. The most common health services older adults used were preventive healthcare, medical treatment, and elderly education, which were the main tasks of primary health care. The primary healthcare system should be improved to ensure the quality and timely delivery of healthcare. China has substantially increased financial investments and introduced favorable policies in order to strengthen the primary healthcare system with core responsibilities in the prevention and management of NCDs during the health-system reform that began in 2009. However, wide gaps in the quality of primary healthcare remain in rural and urban areas [22, 23]. A nationwide cross-sectional survey showed that village clinics and sites in the western region of China had the lowest medication availability for controlling hypertension, which was related to the higher risk of cerebrovascular disease in rural areas [24]. To address disparities in healthcare for older adults, more efforts need to be directed at improving the primary healthcare system and ensuring the quality and timely delivery of healthcare, especially in rural areas.

Public health and general practice services should be improved to reduce disease risk in older adults. Of the top five NCDs of older adults, diabetes and hypertension were incorporated into the National Basic Public Health Service Program. Older adults could obtain standardized community-based disease monitoring and health management through contracted family doctors, previous evidence showed improvements in the control of chronic disease conditions [25]. COPD has become one of the top three highest-ranking NCDs with respect to hospitalization rates for the older population, which will result in appreciable consumption of medical resources. It is essential that population-based COPD prevention strategies be tailored to people with different health risks [26]. Intervertebral disc disease entered the list of the top five NCDs for the first time in 2018, and its prevalence rates in rural areas were higher than in urban areas. If COPD and intervertebral disc disease could be included in basic public health services, it would improve the social cognition, diagnosis, treatment, and health management of diseases.

### The increasing prevalence of NCDs and functional dependence need integrated healthcare systems centered on older adults

Although primary care institutions provide all of the aforementioned services, the public health workers (in charge of public health services) and doctors (in charge of medical treatment) work in separate departments and there are limited mechanisms to facilitate cooperation in providing integrated care for the prevention and treatment of disease and the promotion of health. The fragmentation of clinical care and public health services has created a bottleneck in improving the health of the population in China [15]. Country case studies in Andorra, China, France, and India provided evidence to suggest that the large-scale implementation of integrated care for older people in clinical practice is feasible to recognize the need for a transformation of health and social care systems and services to deliver integrated and person-centered care for older people [27]. To accelerate progress, health policy should seek an optimal match between the demands of aging and the health delivery system and enhance synergy among health financing, public health initiatives, and elderly care services.

The healthcare system should deal with the diverse long-term care and other demands related to aging. Older adults exhibit a considerable willingness to accept home-based services, and it is necessary to form integrated services around their needs. A Long-term Care Medical Insurance (LTCMI) program in Qingdao showed that such a system would not only provide older adults with long-term medical care services but also greatly relieve the pressure on hospitals caused by aging patients [28]. The healthcare system is thus required to shift from cure-seeking medical care that focuses on disease treatment on an organ-specific basis to cure-and-support-seeking medical care with treatments reprioritized to maximize the quality of life for older adults [29].

Our study has some limitations. First, the NHSS is a cross-sectional survey, while social and economic factors usually affect the prevalence of diseases and health service utilization. The effects of social and economic factors on older adults could not be properly evaluated, given the present cross-sectional design. Second, the prevalence of NCDs may have been underestimated in the survey because these data were self-reported and could thus be affected by recall bias. Missed diagnoses of NCD, especially in rural areas with medical care being accessible only with difficulty, may also have led to underestimation. Recall bias may also reduce the impact of health-related behaviors, which also leads to underestimation of the health service utilization and economic burden placed by disease, although the very large sample size in the survey may reduce this risk. Third, NHSS is a national durative survey, but some variables are inconsistent. During the 6 waves NHSSs, some indicators have been partially adjusted in different years, like health service expenditure. And some have been newly added in subsequent surveys, such as ADL. We have included principal survey data related to health care services of old adults as much as possible, but some variables could not be consistent in the 6 waves NHSSs.

## Conclusion

In the process of rapid aging and urbanization in China, there were substantially increased health service demands and economic burdens. The elevated risks of age-related impairments and chronic morbidities, and diverse health service demands are critical public health issues. In addition, China's healthcare delivery system needs to notably strengthen primary healthcare and move towards integrated delivery to improve access and quality of care for older adults. Therefore, the integration of healthcare and social security systems constitutes an adaptive trend in meeting the health service demands for health aging purposes.

## Data Availability

The National Health Service Survey (NHSS) datasets could be accessed with the authorization of the Centre for Health Statistics Information, National Health Commission of the People's Republic of China.
